# Molecular docking, synthesis and biological evaluation of Vascular Endothelial Growth Factor (VEGF) B based peptide as antiangiogenic agent targeting the second domain of the Vascular Endothelial Growth Factor Receptor 1 (VEGFR1D2) for anticancer application

**DOI:** 10.1038/s41392-020-0177-z

**Published:** 2020-06-05

**Authors:** Afsaneh Sadremomtaz, Ameena M. Ali, Foroozan Jouyandeh, Saeed Balalaie, Razieh Navari, Sylvain Broussy, Kamran Mansouri, Matthew R. Groves, S. Mohsen Asghari

**Affiliations:** 1 XB20 Drug Design, Groningen Research Institute of Pharmacy, University of Groningen, 9700 AD Groningen, The Netherlands; 2 Qatar Biomedical Research Institute (QBRI), Hamad Bin Khalifa University (HBKU), Doha, Qatar; 3 Department of Biology, Faculty of Sciences, University of Guilan, Rasht, Iran; 4 Peptide Chemistry Research Center, K.N. Toosi University of Technology, K. N. Toosi University of Technology, Tehran, Iran; 5 University of Paris, Faculty of Pharmacy of Paris, CiTCoM, 8038 CNRS, U 1268 INSERM, F-75006 Paris, France; 6 Medical Biology Research Center, Kermanshah University of Medical Sciences, Kermanshah, Iran; 7 Institute of Biochemistry and Biophysics (IBB), University of Tehran, Tehran, Iran

**Keywords:** Breast cancer, Breast cancer, Structural biology

**Dear Editor**,

Tumor angiogenesis is regulated by the binding of VEGF to receptors (VEGFRs). The major mediator of tumor angiogenesis is VEGFA, more commonly referred to as VEGF. VEGFR1 binds VEGF with higher affinity (10 times) than VEGFR2.^1^ However, the role of the VEGFR1 in VEGF-mediated angiogenesis remains a mystery.

In order to disrupt the VEGFB–VEGFA/VEGFR1D2 interaction as a tool to study the angiogenesis process,^1,2^ the first step was the generation of a second extracellular domain of the VEGFR1. Hence, His-tagged VEGFR1D2 was expressed and purified (Supplementary Fig. S1). The addressed protein could be used in biophysical, biological, and in vivo studies with an appropriate VEGF receptor binding peptide. Such a peptide could be designed and synthesized (Supplementary Figs. S1–S6) and (Supplementary Tables S1 and S2).

The aim of this study was to develop a simplified system to enable the rational design of inhibitor of VEGFB–VEGFA/VEGFR1D2 system (PDB ID:2XAC and 1FLT). Among large surface areas comprising five main interacting regions, a small peptide antagonist could be designed and only act at a single site covering a small surface area with high-affinity interaction. Here, we described designing such a peptide referred to as VGB3 using “protein contact atlas”, “LigPlot” and “PocketQuery”. VGB3 binds and neutralizes VEGFR1D2. A major binding site for VEGFR1D2 binding involves residues 60–67 (the loop region connecting β3 to β4), 62-PDDGL-66, and strand β7 (103–106) 102-ECRP-105 of VEGFB.^1,3^ In addition, to increase peptide half-life in circulation, a *Cys* residue was added to the C terminus to make the disulfide bond. The loop region has more numbers of acidic residues, which are highlighted by LigPlot and PocketQuery, and could be recognized as polar interactions with VEGFR1D2. In good agreement, Iyer et al. reported the similarity of interactions in all three receptor bound complexes of VEGFB–VEGFR1D2, VEGFA–VEGFR1D2, and PlGF–VEGFR1D2 by this acidic stretch of residues. It would be assumed that these polar interactions would play an important role in VEGFB:VEGFR1D2 complex.^1^ Next, the peptide was designed and synthesized by using a solid-phase peptide synthesis procedure. Accordingly, the mimetic decapeptide VGB3 introduced by this sequence; **2NH-Glu1-Cys2-Arg3-Pro4-Pro5-Asp6-Asp7-Gly8-Leu9-Cys10-COOH**.

To gain structural information about the molecular interface of the bound VGB3 peptide, a structural model of VGB3/VEGFR1D2 complex has been generated using the ZDOCK web server. The binding energetic of VGB3/VEGFR1D2 was calculated and the free binding of energy value is −30.819 kcal/mol. The energetic analysis also supported this finding, that is, VGB3 can bind tightly to VEGFR1D2 as compared with free energy of binding for same antagonist target complexes.^1,3^ Analysis of the VGB3:VEGFR1D2 interface Ligplot+ and Pocketquery shows direct interactions between VGB3 and residues within the loop region connecting β3 to β4 and strand β7 of VEGFR1D2 (Fig. 1b). In the VGB3:VEGFR1D2 complex, the residues from this loop region make a total of 14 van der waals interactions with Pro143, Leu204, Phe172, Lys170, Pro173, Leu174, Lys171, Thr206, Glu208, Leu215, and Lys217 of VEGFR-1D2.^3,4^ The most van der Waals contacts were from hydrophobic residues such as Phe172 and Leu204 of VEGFR1D2 to Cys2, Arg3, Cys10, and Leu9 of VGB3. In order to highlight the crucial role of polar interaction in the loop region, Iyer et al. performed the mutation and the results indicated 67% of total interactions of VEGFB (a total of 12 interactions). Detailed comparison of full crystal structure VEGFB:VEGFR1D2 complex (PDB: 2XAC) and present derivative VGB3:VEGFR1D2 complex gives an insight as to why this might be the case. All active residues in interaction VGB3 to VEGFR1D2 are listed in Table S3, which includes mostly a network of van der Waals and polar contacts (Fig. 1a and Supplementary Table S3). Potential polar contacts can also be identified involving amide groups of the VEGFR1D2 surface and carboxyl group of side chains of the peptide.

**Fig. 1:**
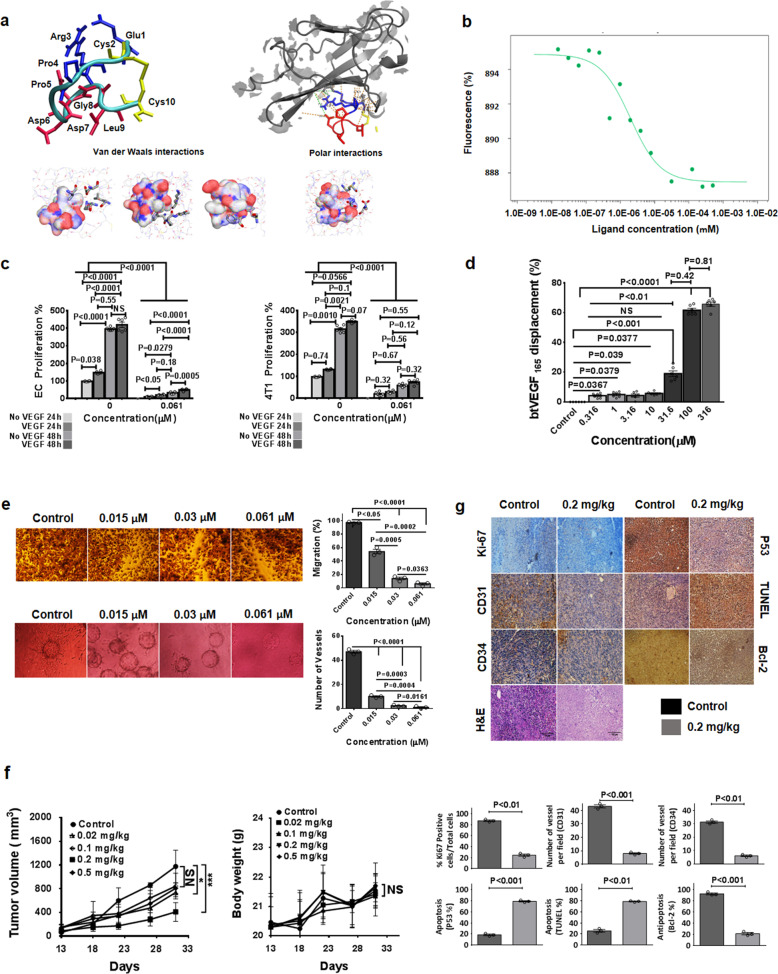
**a** Docking studies. The derived molecular model was created using ZDOCK. The models were created by PEPFOLD3 software, version 9.16, and figures were generated with a UCSF Chimera. Ligplot+ & Pocketquery generated representation of the interaction between VGB3 and VEGFR1D2. The polar and van der Waals interactions are indicated in green and orange, respectively. The Pocketquery results identify side chains of VGB3 residues that may be involved in important interactions (Polar and van der Waals interactions) at the complex interface, as listed in Table S3. **b** MST curve. The result of binding of VGB3 to VEGFR1D2 at a stoichiometry of 1:1. The assay was performed using a fixed concentration of fluorescently labeled VEGFR1D2 and the initial concentration of ligand was 0.25 mM. **c** The effect of VGB3 on VEGF-stimulated cell proliferation. The effect of VGB3 on cell proliferation was determined on endothelial cells as well as 4T1 tumor cells. The cells with (dark grey) and without VEGF (light grey) were treated with various concentration ranges (0–0.061 μM) of VGB3 and then an MTT assay was performed to measure cell viability at two time points (24 and 48 h). Data points are mean ± SEM, obtained by Prism 6. Unpaired two-tailed *t*-test (to compare the differences between No-VEGF and VEGF in each concentration) and one-way ANOVA with Tukey multiple comparison; *n* = 6, NS no significant. **d** ELISA-based displacement assay. Bars represent mean ± SEM, obtained by Prism 6; one-way ANOVA with Tukey multiple comparison. **e** VGB3 inhibits angiogenesis in vitro. VGB3 inhibits migration of wounded HUVEC monolayers compared with the control. VGB3 decoy assessment in HUVEC Cytodex 3D bead sprouting assay which followed by embedding into the collagen gel in the presence of VEGF (0.75 pM) as stimulator following exposure to increasing concentrations of VGB3. Analytical results of mean data are shown for each concentrations. Bars represent mean ± SEM, obtained by Prism 6. Unpaired two-tailed *t*-test and one-way ANOVA with Tukey multiple comparison, NS no significant. **f** VGB3 inhibits 4T1 metastatic breast cancer growth in vivo. Significant inhibition of tumor growth occurred in animals treated with VGB3 when compared with control. Data points are mean ± SEM, obtained by Prism 6 in two-way ANOVA statistical analysis; *n* = 6. The differences between VGB3 treatments and PBS controls are demonstrated; *P ≤ 0.05 (0.02 mg/kg of VGB3 to PBS control), ***P ≤ 0.001 (0.2 mg/kg of VGB3 to PBS control), no significant (0.1 mg/kg and 0.5 mg/kg of VGB3 to PBS control). The average body weight of each group was measured until the size of the tumor in the control animals reached the endpoint of the study (from day 13 till 31), then presented as mean ± SEM. The data indicated no significant (NS) differences between VGB3 treatments and PBS controls in two-way ANOVA statistical analysis. **g** Mechanistic basis for VGB3-mediated tumor inhibition in vivo. Data were analyzed by ImageJ, and visualized as columns bars using Prism 6. Results shown are representative or mean ± SEM (*n* = 6). The differences between VGB3 treatments (0.2 mg/kg) and PBS controls were assessed by unpaired two-tailed *t*-test

Subsequently, to verify the ability of VGB3 to form a complex with VEGFR1D2, microscale thermophoresis (MST) measurements were performed using a VEGFR1D2-labeled with VGB3 at increasing concentrations. As well as measuring the K_d_ between VGB3 and its receptor this functional test will also confirm whether the refolded protein VEGFR1D2 assumes the correct conformation. Fluorescently labeled VEGFR1D2 purified and titrated as 1:1 with VGB3. The curve for VGB3 vs VEGFR1D2 exhibited a dissociation constant (K_d_) of 1.96 ± 0.69 μM (Fig. 1b). It should be noted that this binding constant is 40-fold lower than the K_d_ (K_d_ = ~50 nM) of VEGFA to the entire extracellular portion of VEGFR1. Previously, a tight association between VEGFR1 D2 and VEGF has also been reported (113pM).^4^ This difference between VGB3 and VEGF in affinity indicates that multiple regions of VEGF are required in the binding of VEGFR. However, the single digit micro molar affinity measured in MST indicates that the dominant portion of the binding interaction is mimicked by the VGB3 peptide.

Moreover, VEGFR1 binding property of VGB3 was confirmed by its ability to inhibit the proliferation of 4T1 mammary carcinoma cell line, which highly express VEGFR1.^1,2,5^ VGB3 could inhibit VEGF165 (0.75 pM)-stimulated proliferation of 4T1 mammary carcinoma cells, which express VEGFR1, with the half maximal cell growth inhibitory concentration (IC50) of 30 nM (****P < 0.0001) (Fig. 1c and Supplementary Fig. S7). This value is 6-fold lower than the one for the VEGFR1D2 fragments those which can inhibit VEGF165-stimulated proliferation of human umbilical vein endothelial cells with an IC50 of ~180 nM.^3^ These results suggest that VGB3 is more potent than other peptides reported by those which have been designed from the same ligand.^2,4,5^ Similarly, in other investigations, the VEGFR-1-binding ELISA-based assay developed and allows us to approve the inhibitory effect VGB3 on the VEGF-A/VEGFR-1 interaction. VGB3 had significantly increased potency, up to 63% displacement of btVEGF165 (100 pM) detected at 100 μM as compared to the potential antiproliferative potency of VGB3 to inhibit 50% VEGFA (0.75 pM)-induced proliferation of the HUVEC as well as 4T1 mammary carcinoma cell lines at 30 nM (Fig. 1c–e and Supplementary Fig. S7). By comparison, VGB3, with an IC50 value of 30 nM, more effectively inhibited endothelial cell proliferation than other peptides that we recently reported based on the binding regions of VEGF-B.^2,4,5^ In good agreement, our study showed that VGB3 is more potent than those of clinically used antiangiogenic cancer drugs such as Bevacizumab in endothelial cell culture conditions.^2,4,5^

The antitumor effects of VGB3 were examined in the murine 4T1 mammary carcinoma tumor model (MCT), which is a well-established VEGFR1-dependent model.^2,5^ Following intravenous administration, the in vivo tumor regression was performed and the inhibitory effects of VGB3 on tumor growth and angiogenesis were definitely higher (0.2 mg/kg, once in every 2 days) than those which have reported by same background antiangiogenic drugs such as bevacizumab (2 mg/kg, twice per week) (Fig. 1f). In good agreement with immunohistochemistry analysis (as evidenced by decreased CD31, CD34, Ki67 expression, Bcl-2 and increased TUNEL staining and p53 expression), suggest that the antitumor efficacy of VGB3 is closely correlated with its antiangiogenic effect and with the induction of apoptosis in a xenograft mouse model. On the basis of these results, VGB3, through neutralization of VEGFR-1, that could be indirectly involved in impairing many angiogenesis signaling pathways, which lied at downstream of VEGFR1 (Fig. 1g).

Our results confirm that targeting VEGFR1 is a reliable approach for inhibition of tumor growth and angiogenesis and VGB3 can be a candidate for antiangiogenic therapy.

## Supplementary information

Supplementary Material

## References

[1] Iyer S, Darley PI, Acharya KR (2010). Structural insights into the binding of vascular endothelial growth factor-B by VEGFR-1D2 recognition and specificity. J. Biol. Chem..

[2] Sadremomtaz A (2018). Dual blockade of VEGFR1 and VEGFR2 by a novel peptide abrogates VEGF driven angiogenesis, tumor growth, and metastasis through PI3K/ AKT and MAPK/ERK1/2 pathway. Biochim. Biophys. Acta Gen. Subj..

[3] Wiesmann C (1997). Crystal structure at 1.7 A resolution of VEGF in complex with domain 2 of the Flt-1 receptor. Cell.

[4] Wang L (2017). Identification of peptidic antagonists of vascular endothelial growth factor receptor 1 by scanning the binding epitopes of its ligands. J. Med. Chem..

[5] Sadremomtaz A (2018). Suppression of migratory and metastatic pathways via blocking VEGFR1 and VEGFR2. J. Recept. Signal Transduct. Res..

