# Supplementary material for: Molecular docking, synthesis and biological evaluation of Vascular Endothelial Growth Factor (VEGF) B based peptide as antiangiogenic agent targeting the second domain of the Vascular Endothelial Growth Factor Receptor 1 (VEGFR1D2) for anticancer application
Source: Signal Transduct Target Ther. 2020 Jun 5;5:76. doi: 10.1038/s41392-020-0177-z (PMC7272640; doi:10.1038/s41392-020-0177-z)
Supplement: Supplementary file 1 — Supplementary Material [file 41392_2020_177_MOESM1_ESM.docx]

**Supplementary information:**

**Molecular docking, synthesis and biological evaluation of Vascular Endothelial Growth Factor (VEGF) B based peptide as anti-angiogenic agent targeting the second domain of the Vascular Endothelial Growth Factor Receptor 1 (VEGFR1D2) for anticancer application**

Afsaneh Sadremomtaz^1^, Ameena M. Ali^1,2^, Foroozan Jouyandeh^3^,  Saeed Balalaie^4^, Razieh Navari^4^, Sylvain Broussy^5^, Kamran Mansouri^6^, Matthew R Groves^1*^, S.Mohsen Asghari^3,7*^

**Affiliations**

^1^ XB20 Drug Design, Groningen Research Institute of Pharmacy, University of Groningen, 9700 AD Groningen, The Netherlands.

^2^ Qatar Biomedical Research Institute (QBRI), Hamad Bin Khalifa University (HBKU), Doha, Qatar.

^3^ Department of Biology, Faculty of Sciences, University of Guilan, Rasht, Iran.

^4^ Peptide Chemistry Research Center, K.N. Toosi University of Technology, K. N. Toosi University of Technology, Tehran, Iran.

^5^ UMR 8638 CNRS, Facultéde Pharmacie de Paris, UniversitéParis Descartes, Sorbonne Paris Cité, 4 Avenue de l’Observatoire, Paris 75006, France.

^6^ Medical Biology Research Center, Kermanshah University of Medical Sciences, Kermanshah, Iran.

^7^ Institute of Biochemistry and Biophysics (IBB), University of Tehran, Tehran, Iran.

* Corresponding authors:

Pro. Matthew Groves: E. Mail: m.r.groves@rug.nl. Phone: [+31 50 36 33305](tel:+31503633305)

Dr. S. Mohsen Asghari: E. Mail: sm_asghari@guilan.ac.ir. Phone: +989122432070

This supplementary data file contains:

• Supplementary Figure 1-7

• Supplementary Table 1-3

• Supplementary Methods

• References

**Supplementary Figures:**

**a**


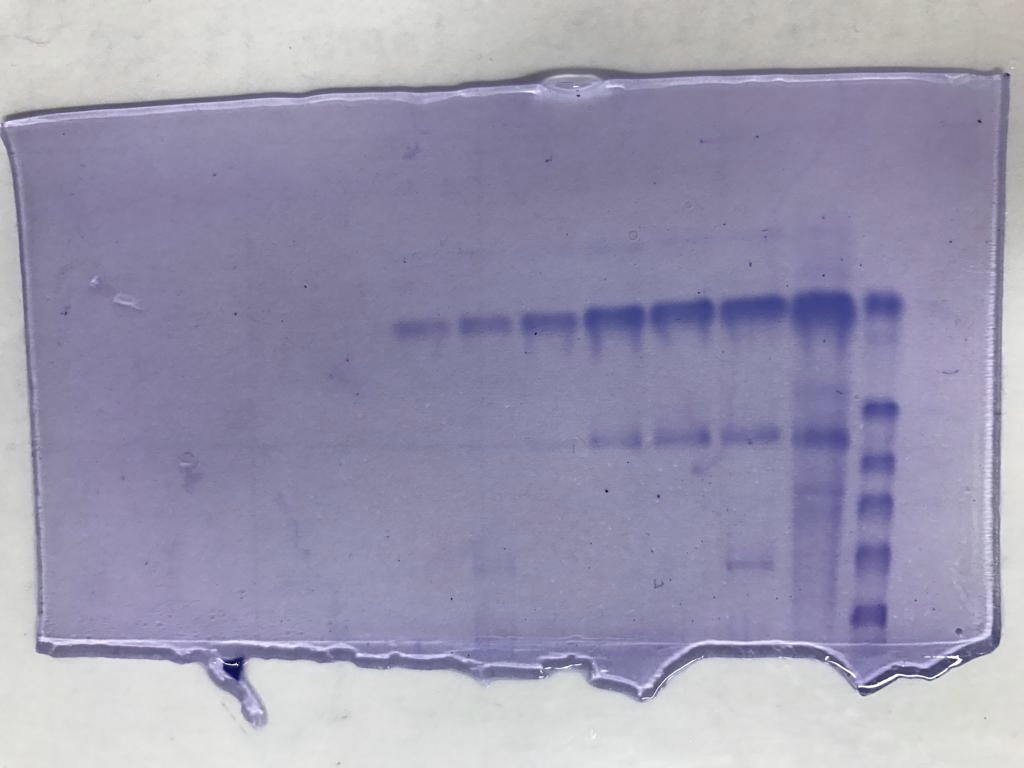

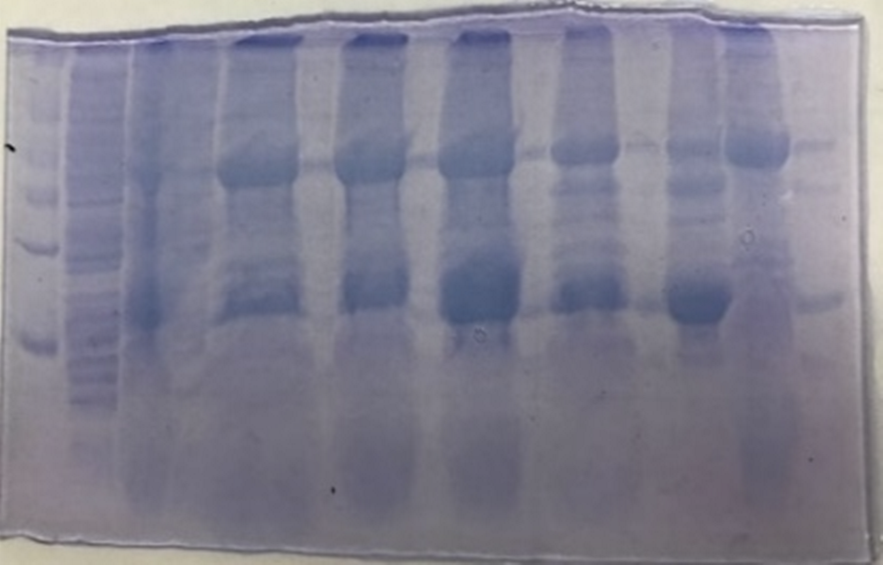


**66 kDa**

**36 kDa**


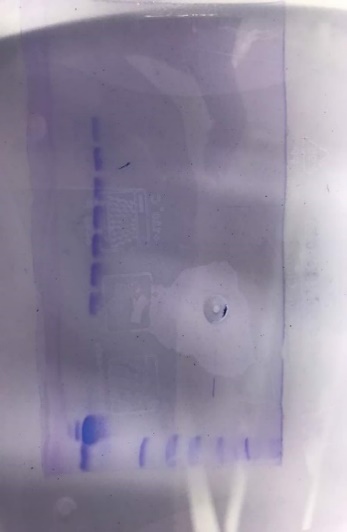


**29 kDa**

**20 kDa**

**14.2 kDa**

**6.5 kDa**

**M**

**M**

**M**

**b**


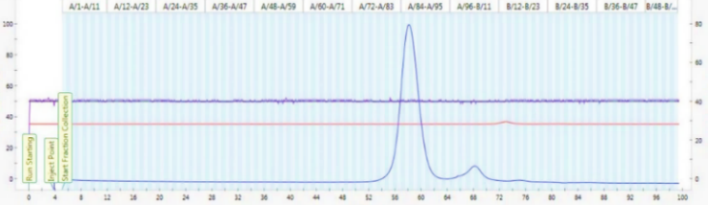


**Time (min)**

**Conductivity (mS/cm)**

**λ(280 nm) (mAU)**

Figure S1: Protein purification and SDS-PAGE analysis of the recombinant VEGFR1D2. (a) Expression in *E. coli* BL21 Codonplus (DE3) RIL strain. *E. coli* crude extract pellet and supernatant followed by 5 times washing, respectively.  Representative the fold eluted His-tagged VEGFR1D2 fractions after nickel-nitrilotriacetic acid (Ni^2+^–NTA) purification and compared to unfolded protein (second lane). (b)  The size exclusion chromatography (SEC)-peak fractions obtained in the chromatogram and concentrated. M, molecular weight marker. The chromatogram revealed a single peak in the presence of Tris 50 mM and NaCl 250 mM.

Figure S2: The Synthetic pathway for the synthesis of cyclopeptide sequence. The general method for the synthesis of cyclopeptide based peptide using SPPS.


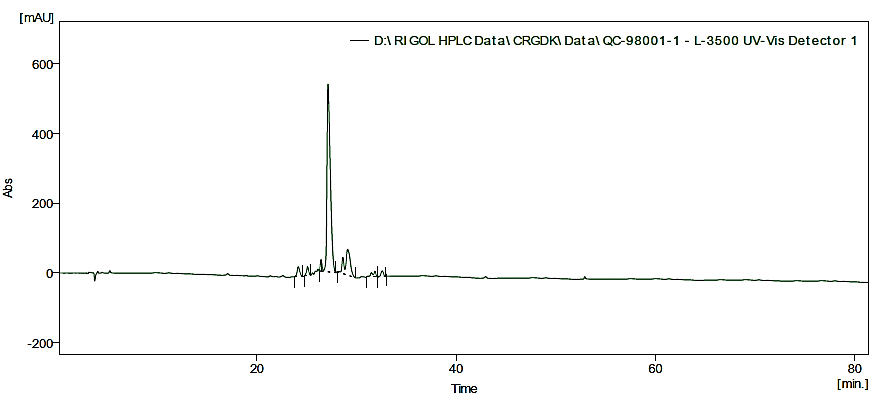


  Figure S3. HPLC chromatogram of linear decapeptide 1

Figure S4.  LC-MS of linear decapeptide 1


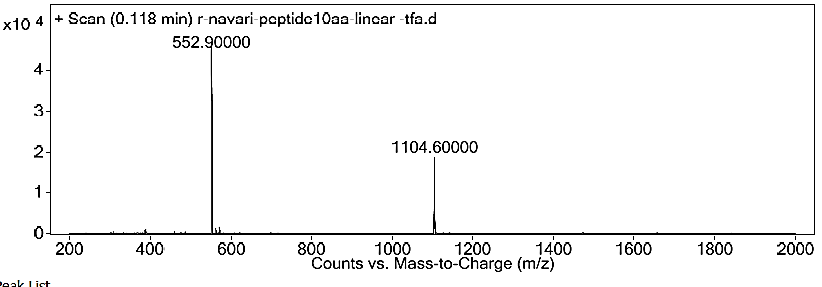


Figure S4: LC-MS of linear decapeptide **1**


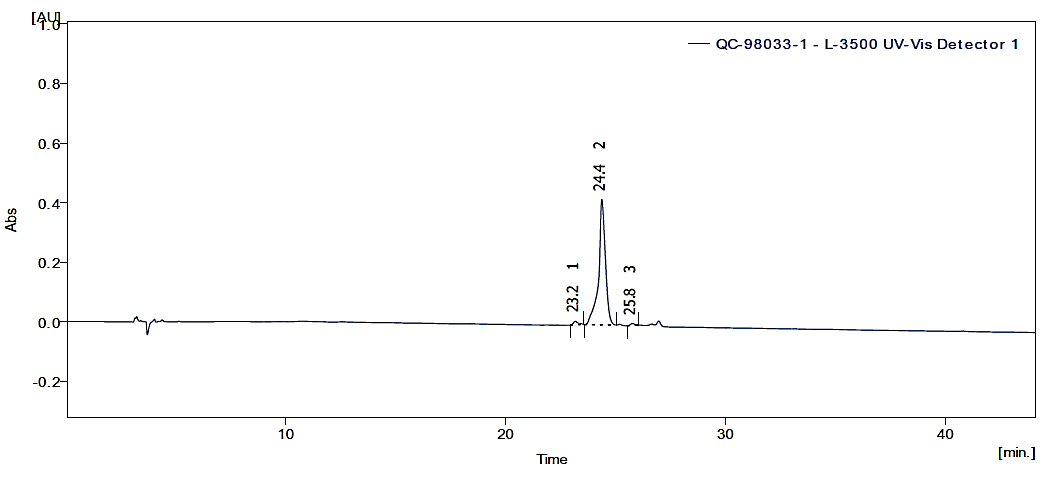


Figure S5. HPLC chromatogram of cyclopeptide


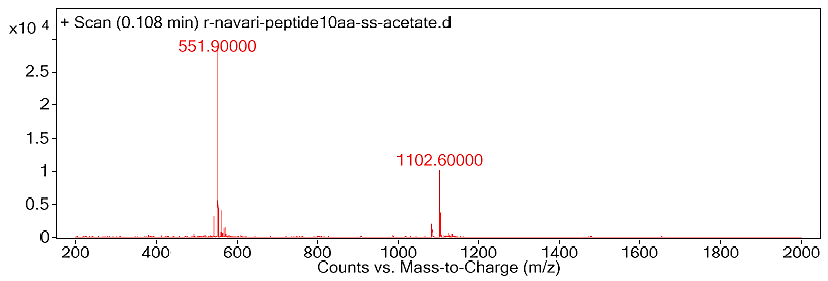


Figure S6. LC-MS of cyclopeptide

Figure S7. The effect of VGB3 on VEGF-stimulated cell proliferation. The effect of VGB3 on cell proliferation was determined on endothelial cells as well as 4T1 tumor cells. The cells with (dark grey) and without VEGF (light grey) were treated with various concentration ranges (0 - 0.061 μM) of VGB3 and then an MTT assay was performed to measure cell viability at two time points (24 and 48 h). Data points are mean ± SEM, obtained by Prism 6. Unpaired two-tailed t-test (to compare the differences between No-VEGF and VEGF in each concentration) and One-way ANOVA with Tukey multiple comparison; n=6, NS; no significant.

**Supplementary Tables:**

Table S1:Peptide purification

| Entry | Flow (ml/min) | Time (min) | A% | B% |
| --- | --- | --- | --- | --- |
| 1 | 10 | 0 | 95 | 5 |
| 2 | 10 | 5 | 95 | 5 |
| 3 | 10 | 65 | 45 | 55 |
| 4 | 10 | 110 | 0 | 100 |

Table S2: Analytical RP-HPLC separation

| Entry | Flow (ml/min) | Time (min) | A% | B% |
| --- | --- | --- | --- | --- |
| 1 | 1 | 0 | 95 | 5 |
| 2 | 1 | 5 | 95 | 5 |
| 3 | 1 | 45 | 55 | 45 |
| 4 | 1 | 100 | 0 | 100 |

Table S3: Intermolecular contacts at VGB3:VEGFR1D2 interface

| **VGB3** | **VEGFR1D2 Polar contacts** | **Distance (A^⸰^)** | **VGB3** | **VEGFR1D2 Van der Waals contacts** | | **Distance (A^⸰^)** |
| --- | --- | --- | --- | --- | --- | --- |
| Glu 1 | Glu 141 | 4 | Glu 1 | | Pro 143 | 3.7 |
| Arg 3 | Asn 219 | 3.5 | Cys 2 | | Leu 204  Phe 172 | 6.8  7 |
| Asp 7 | Lys 217 | 7 |  |  |  |  |
|  |  |  | Cys 10 | | Phe 172 | 8.3 |
|  |  |  | Leu 9 | | Phe 172 | 8.2 |
|  |  |  | Arg 3 | | Leu 204 | 6.8 |
|  |  |  | Pro 4 | | Lys 170  Pro 173  Leu 174  Lys 171 | 6.7  4.7  5.9  6.1 |
|  |  |  |  |  |  |  |
|  |  |  |  |  |  |  |
|  |  |  |  |  |  |  |
|  |  |  | Pro 5 | | Thr 206  Glu 208  Leu 215  Lys 217 | 8.4  9.1  10.2  9 |
|  |  |  |  |  |  |  |
|  |  |  |  |  |  |  |
|  |  |  |  |  |  |  |

**Supplementary Methods:**

2.1 Reagents:

Commercially available materials were used without further purification. 2-Cl-Trt resin and the amino acids were purchased from Iris Biotech Company,  *N, N*-Diisopropylethylamine (DIEA) from Sigma-Aldrich. Organic Solvents (DMF, DCM, MeOH, and CH_3_CN) were purchased from Merck. MonolithNT.115 instrument was from NanoTemper Technologies, Germany. RP-HPLC quality acetonitrile and water were used for RP-HPLC analyses and purification. FITC-secondary anti-rabbit (Ab6717), anti-CD31 (Ab32457), anti-CD34 (Ab81289), anti-Ki67 (Ab15580), anti-P53 (Ab131442), and anti-Bcl-2 (Ab59348) were from Abcam, Cambridge, UK; and TUNEL assays were performed using an in situ Cell Death Detection Kit POD (Roche Diagnostic GmbH, Germany).

2.2 VEGFR1D2; Transformation, Expression, Refolding, and purification:

The expression plasmid of VEGFR1D2 was a kind gift from Luca D’ Andrea, Istituto di Biostrutture e Bioimmagini, CNR, Napoli, Italy. The identity of the insert in the resulting recombinant plasmid was confirmed by DNA sequencing. *E. coli* BL21 Codon Plus (DE3) RIL cells (Stratagene) were transformed with the pETM11-VEGFR1D2 plasmid and grown in the presence of antibiotics (50 μg.ml^-1^ kanamycin and 33 μg.ml^-1^chloramphenicol) at 37^⸰C^, under shaking, until reaching an OD600  of 0.7/0.8. They were then induced with 0.7 mM IPTG. After 4–5 h the cells were harvested by centrifugation, the pellet was dissolved in 50 mM Tris-HCl, pH=8 and sonicated. The bacterial lysate was centrifuged. The soluble fraction and the pellet were analyzed by SDS-PAGE. The recombinant His-tagged VEGFR1D2 was solubilized from inclusion bodies in 50 mM Tris-HCl, 10 mM imidazole, 8M urea, pH=8. The cleared lysate was loaded on Ni2+-NTA resin (1h, 4^⸰C^) in the presence of 300 mM NaCl. The His-tagged protein was refolded by equilibrating the resin in 50 mM Tris-HCl, 10 mM imidazole, 300 mM NaCl, pH 8 with decreasing concentrations of urea and protein was eluted using an increasing imidazole concentration from 10 to 300 mM. The His-tagged VEGFR1D2 was incubated against Glutathione (3 mM reduced/0.3 mM oxidized) to 1h. Then the protein was concentrated by the Amicon Ultra system (3000 MWCO, Millipore) and purified by size exclusion chromatography (SEC) using an S75 column (GE Healthcare) equilibrated in 50 mM Tris-HCl and 250 mM NaCl, pH 7. Finally, the SEC peak fractions were concentrated to 1.63 mM using the small Amicon Ultra system (14000 MWCO, Millipore).

2.3 peptide design:

The design of peptide antagonists was based on the co-crystallized structure of VEGF-A with VEGFR-1 D2 (PDB code 1FLT) and VEGF-B with VEGFR-1 D2 (PDB code 2XAC), using “protein contact atlas”, “LigPlot” and “PocketQuery” software.

2.4  General Information to Peptide Synthesis:

Peptides were manually using standard Fmoc solid-phase peptide synthesis chemistry. The amino acids were used: Fmoc-Cys(Trt)-OH, Fmoc-Leu-OH, Fmoc-Gly-OH, Fmoc-Asp(*t*Bu)-OH, Fmoc-Pro-OH, Fmoc-Arg(Pbf)-OH, Fmoc-Glu(*t*Bu)-OH.

**General Method  for the synthesis of linear decapeptide H-Glu-Cys-Arg-Pro-Pro-Asp-Asp-Gly-Leu-Cys-OH (1):**

The decapeptides were synthesized using known Fmoc solid-phase peptide synthesis Strategy. The synthesis of the peptides was carried out using 2-chlorotrityl chloride resin (1.0 mmol/g) following standard Fmoc strategy. At first, the resin was swelled with DCM (3×10 ml) for 30 min. Then Fmoc-Cys(Trt)-OH (0.586 g, 1.0 mmol) was attached to the 2-CTC resin (1.0 g) with DIPEA (1.36 ml, 8 mmol) in anhydrous DMF (10 ml) at room temperature for 3 h. After filtration, the remaining trityl chloride groups were capped by a solution of DCM/MeOH/DIPEA (20:2.4:1.2, 23.6 ml) for 30 min. The resin was filtered and washed thoroughly with DMF (3× 10 ml). The loading capacity was determined by weight after drying the resin under vacuum and was 1.0. The resin-bound Fmoc-amino acid was washed with DMF (3× 10 ml) and treated with 25% piperidine in DMF (15 ml) for 30 min and then it was washed with DMF (3× 10 ml). Then a solution of Fmoc-Leu-OH (0.708 g, 2.0 mmol), TBTU (0.64 g, 2.0 mmol), DIPEA (0.6 ml, 3.5 mmol) in 10 ml DMF was prepared and added to the resin-bound free amine and shaken for 45 min at room temperature. After completion of the coupling, the resin was washed with DMF (3× 10 ml). The coupling was repeated as the same method for other amino acids of the sequence. In all cases, the coupling was confirmed using Kaiser to detect the presence or absence of free the primary amino groups. Fmoc concentration determination was done using UV spectroscopy. After completion of all couplings, the resin was washed with DMF (3×10 ml). The produced decapeptide (1) was cleaved from the resin by treatment with TFA (1%) in DCM (100 ml) and neutralization with pyridine (4%) in MeOH (50 ml). The solvent was evaporated under reduced pressure and precipitated in water. Then, 1 g of dry protected decapeptide was treated with TFA/TES/H_2_O/MeOH (95%: 2%: 1.5%: 1.5%) for 2-3 h at rt. The yield was 70% (0.773 g of decapeptide 1).

**General Method  for the synthesis of cyclopeptide (2)**

The fully deprotected peptide (1) was dissolved in H_2_O or H_2_O/acetonitrile. In the following the solution was buffered to pH = 9 with ammonium acetate 10% was added subsequently and the mixture was stirred for 6 h at room temperature. Subsequently, the mixture was lyophilized. Finally, the cyclopeptide (2) was analyzed by HPLC and Mass (ESI). (yield: 98%)

Peptide Synthesis:

The cyclopeptide was synthesized following the standard Fmoc-Solid Phase Peptide Synthesis (SPPS) procedure on 2- chlorotrityl resin and using TBTU as a coupling reagent (Figure S1). At first, Fmoc-Cys(Trt)-OH was loaded to the surface of resin using diisopropylethylamine (DIEA) and after capping with MeOH other Fmoc-protected amino acids were added to the peptide sequence using TBTU as coupling reagent.

Coupling reactions were performed using Fmoc-amino acid/TBTU /DIEA/. Fmoc groups were deprotected by treating the resin using 25% piperidine in DMF. To confirm the coupling of the amino acids Kaiser test was performed. After the successive addition of the 10 amino acids in the sequence, the desired fully protected decapeptide on the surface of resin A was obtained. To access linear decapeptide based peptide (1), the following steps were made a) the protected peptide A was cleaved from the resin surface using 1% TFA and compound B was formed. b) Then, the final deprotection was done using reagent K (TFA/TES/H_2_O/MeOH (95%: 2%: 1.5%: 1.5%). c) To access the desired cyclopeptide (2), cyclization of the fully deprotected peptide was performed in 500 ml of  H_2_O or H_2_O/acetonitrile (for 200 mg of linear peptide) with air oxidation. The solution was buffered to pH = 9 with ammonium acetate 10% was added subsequently. Cyclization was usually complete within 6 hr as shown by HPLC (high-performance liquid chromatography) control. The solvents were then evaporated and the peptides were lyophilized. Yields = 98 d) The purification of the peptide was done using preparative HPLC (Column C-18, Eurospher 100, 7 μm).

The structure of linear decapeptide (1) was approved using Mass(ESI). (Scheme) Also, the structure of cyclopeptide (2) was approved using Mass(ESI). (Scheme)

HPLC analysis found that linear decapeptide (1) was obtained in 97 %< purity (t_R_: 27.13 min). Mass (ESI): C_43_H_69_N_13_O_17_S_2_ *m/z* = [M+H]^+^ Found 1104. HPLC analysis found that cyclopeptide (2) was obtained in 96 %< purity t_R_: 24.38 min). Mass (ESI): C_43_H_67_N_13_O_17_S_2_  *m/z* = [M+ H]^+^ Found  1102.

2.5 High Performance Liquid Chromatography:

The samples were dissolved in solvent A for liquid chromatography. The mobile phase for all HPLC purifications consisted of solvent A (Acetonitrile/Water (70/30)) and solvent B (NaH_2_PO_4_/Water (10mM)) and separations were performed on an HPLC system (Knauer, Germany) equipped with a pump 1800 (Knauer, Germany), UV detector 2500 (Knauer, Germany) (Table S1). Peptide purification was performed at a preparative scale using ODS-C18 column (120 mm × 20mm, 10 μm). The flow rate was set to 10 mL.min^−1^ and The employed elution program started at 95 % A and remained at this point for 5 min before changing to 45 % of solvent A over 65 min. The elution profile was monitored via UV absorbance at 210 nm and peptides were collected manually according to their absorbance at 210 nm.

Analytical RP-HPLC (Rigol, China) separation was carried out using a Agilent C18 column (250 mm × 4.6 mm, 5 μm, 100 Å) at a flow rate of 1 mL min^−1^ and the mobile phase for consisted of solvent A [TFA/Water (0.1%)] and solvent B [Acetonitrile/Buffer A (80/20)] and separations were performed on an HPLC system (Rigol, China) equipped with a pump 1000 (Rigol, China), UV detector 2500 (Rigol, China) (Table S2). The employed elution program started at 95 % A and remained at this point for 5 min before changing to 55 % of solvent A over 45 min. at 1% min^-1^.  The elution profile was monitored via UV absorbance at 218 nm and peptides were collected manually according to their absorbance at 218 nm.

**Peptide 1:** H-Glu-Cys-Arg-Pro-Pro-Asp-Asp-Gly-Leu-Cys-OH

HPLC analysis found that decapeptide (1) was obtained in 91 %< purity (t_R_: 27.13 min). Mass (ESI): C_43_H_69_N_13_O_17_S_2_ *m/z* = [M+H]^+^ Found 1104.

**Peptide 2:** Cyclopeptide

HPLC analysis found that cyclopeptide (2) was obtained in 96 %< purity (t_R_: 24.38 min) (Figure S2 and S4)

Mass (ESI): C_43_H_67_N_13_O_17_S_2_  *m/z* = [M+ H]^+^ Found  1102.

2.6 Mass spectrometry analysis:

LC-MS analyses were performed by a Agilent Triple Quadrupole LC/MS 6410 Diode array detector , ALS, TCC, Bin pump and Degasser: 1200 series. The MS instrument was operated at the following settings: Drying gas: Nitrogen (300 ^o^C); source voltage, 3.5 kV, Nebulizing gas pressure: 50 PSI, Ionization mode: Electrospray. Product ions were then scanned and monitored (Figure S3 and S5).

2.7 Molecular dynamic:

The ZDOCK webserver was used to generate a model of the VGB3:VEGFR1D2 complex. The crystal structure of VEGFB available from the PDB (PDB code 2C7W) and VEGFB in complex with VEGFR1D2 from the PDB (PDB code 2XAC) were used as template. The models were created by PEPFOLD3 software, version 9.16, and figures were generated using UCSF Chimera. The Protein Contacts Atlas ([https://www.mrc-lmb.cam.ac.uk/rajini/views/matrix.html](https://www.mrc-lmb.cam.ac.uk/rajini/views/matrix.html?allchains=2XAC_A-X-B-C-&variant=no&cutoff=0.5&tab=chord&type=0&displayMode=pairSticks&chains=A,A&pdb=2XAC_A&mychain=A&colorless=1&color1=cyan&color2=magenta)) was used to predict non-covalent contacts in the VEGFB:VEGFR1D2 complex. Thereafter, the interaction pattern in the VGB3:VEGFR1D2 complex was visualized using UCSF chimera, Pocketquery (http://pocketquery.csb.pitt.edu/) and LigPlot^+^ (https://www.ebi.ac.uk/thornton-srv/software/LIGPLOT/).

2.8 Microscale thermophoresis (MST):

The binding affinity of peptide to its cognate receptor was measured by Microscale thermophoresis (MST) on a Nanotemper Monolith NT.115 instrument (Nanotemper Technologies GmbH).  Purified VEGFR1D2 was freshly labeled with the Monolith His-Tag RED-tris-NTA labeling dye according to the supplied protocol (Nanotemper Technologies, GmbH). The labeled protein was concentrated using a PES centrifugation filter (3 kDa cutoff; VWR). Measurements were done in MST buffer (50 mM Tris, 250 mM NaCl, pH=7) in standard capillaries (K002; Nanotemper Technologies GmbH). The final concentrations of either labeled protein in the assay were 25 nM. The ligands (VGB3) was titrated in 1:1 dilutions following manufacturer’s recommendations and starting from 0.25 mM. All binding reactions were incubated for 10 min at room temperature followed by centrifugation at 20,000 g before loading into capillaries. All measurements were performed in triplicate using automatically assigned 20% LED and 60% MST power; Laser On-time was 30 sec and Laser Off time was 5 sec.

2.9 VEGFR1 direct binding assay:

A white flat-bottom high-binding 96-well plate (COSTAR 3922) was coated with human extracellular domains (ECD) of VEGFR1D2 (R&D Systems, Abingdon, UK) in PBS (20 ng/well, 5 nM) overnight at 4 °C. The plate was washed twice with 200 μL of wash buffer (PBS containing 0.1% (v/v) Tween 20) and treated with 200 μL of blocking buffer (PBS containing 3% (w/v) BSA) at 37 °C for 2 h, followed by two washes with wash buffer. Volumes of 50 μL of peptide VGB3 solution at twice the desired final concentration in PBS were added in triplicate wells and the plate was kept at 37 °C for 45 min. A solution of btVEGF-A_165_ (R & D Systems, Abingdon, UK) at twice the desired final concentration (100 pM) in 50 μL PBS was added. After 2 h incubation, the plate was washed two times with wash buffer. 100 μL of Streptavidin-Horseradish Peroxidase (Amersham, Pittsburgh, PA, USA) diluted 1:8000 in PBS were then added to each well to detect the btVEGFA_165_ bound to the ECD of VEGFR1. After 45 min incubation at 37 °C in the dark, the plate was washed two times with wash buffer. Volumes of 100 μL of Supersignal West Pico Chemiluminescent Substrate (Pierce, Rockford, IL, USA) were finally added and the chemiluminescence was quantified with a TECAN spectrophotometer plate-reader. The percentages of displacement were calculated by the following formula: 100×[1 – (S − NS)/(MS – NS)] where S is the signal measured, NS is the nonspecific binding signal defined as the signal measured in the absence of coated receptor on the microplate, and MS is the maximum binding signal obtained with btVEGF-A_165_ without the peptide VGB3.

2.10 Cell proliferation assay:

2× 10^3^ HUVEC and 4T1 cell-line were cultured in DMEM medium supplemented with 10% FBS at 37 °C with 5% CO2 in 96-well plate. After 24 h, The cells were incubated in the media supplemented with 2% FBS, different concentrations (0, 0.015, 0.03, 0.061 μM) of VGB3 in the presence of (0.03 μg.ml^-1^) VEGF-A (Sigma, St. Louis, MO) for 24 and 48 respectively. Then, cell proliferation was quantified by 3-(4,5–dimethylthiazol-2-yl)-2 ,5-diphenyl tetrazolium bromide (MTT) (Sigma, St. Louis, MO) solution (5 mg/mL) in PBS (pH 7.4) was added to each well and the plates were incubated in the dark at 37 °C for 4 h. Then the insoluble purple formazan product was dissolved by dimethyl sulfoxide (DMSO). Absorbance was measured at 570 nm using an ELISA plate reader. The assay was performed in triplicate.

2.11 Wound healing assay

3 × 10^3^ HUVECs were grown to confluence in 96-well plate. The monolayer was mechanically wounded using a sterile pipette tip followed by washing with PBS. The cells were incubated in the serum-starved DMEM medium containing 2% FBS and different concentrations (0, 0.015, 0.03, 0.061 μM) of VGB3 in the presence of (0.03 μg.ml^-1^) VEGF-A for 24 h. Then HUVECs were washed with PBS two times and fixed using 4% paraformaldehyde at room temperature. After staining the cells with Giemsa, photographs were taken under a microscope (scale bar: 20 μm; Olympus BX-51, Japan) immediately and after 24 h incubation at the same location. Data are expressed as the average percentage wound closure for each group (n=6) of initial migrated cells at time 0 (T0), or as the percentage inhibition compared with a time-matched control. The wound area (the open area of the scratch) was quantified using ImageJ: (1 - (wound area at 24 h / wound area at 0 h))×100.

2.12 In Vitro Sprout Formation Assay:

The fibrin or collagen sprouting angiogenesis assay was performed as previously described. Type I collagen was obtained as previously reported. HUVECs were allowed to attach to Cytodex microcarrier beads (Amersham Pharmacia Biotech, Sweden) by incubation in DMEM medium with 10% FBS for 4 h at 37 °C in 5% CO2 (60 μL of 50 g/mL Cytodex beads were coated with 400 μl of HUVECs). EC-covered beads were embedded into the collagen gel under sodium bicarbonate conditions (Sigma, St. Louis, Missouri, USA), distributed in 96-well plates (100 μl/well), and placed in a 37 °C, 5% CO2 incubator for 30 min. Then DMEM supplemented with 2% FBS with a range of concentrations (0, 0.015, 0.03, 0.061 μM) of VGB3 in the presence of (0.03 μg.ml^-1^) VEGF-A were added on top of the collagen gel. After 24 h, angiogenesis was monitored microscopically. All the capillary-like structures were photographed using an inverted microscope (Olympus BX-51, Japan) with a scale bar of 20 μm, and the number of sprouts grown on beads was quantified by ImageJ software. The assays were performed in triplicate.

2.13 *In vivo* antitumor efficacy evaluation:

Female BALB/c mice (5–7 weeks) were purchased from the Laboratory Animal Center of the Iran Pasteur Institute and maintained under standardized environmental conditions: 12 h light-dark phases, with free access to food and water. For treatment with VGB3, Tumor cells (4T1; 1×10^6^ cells/500 μl or 1×10^5^ cells/50 μl) were injected subcutaneously into the right flanks of mice (*n*=3–5). To generate the metastatic model, 4T1 tumor models were sterilized, excised from the breast cancer-bearing BALB/c mice, cut into pieces of <0.3 cm3, and subcutaneously implanted into the animals' right flanks under ketamine (100 mg/kg, i.p.) and xylazine (10 mg/kg, i.p.) anesthesia. Animals carrying tumors of size ~100mm3 were randomized to 6 mice/group (*n*=6). The treatment groups received 0.02, 0.1, 0.2 or 0.5 mg/kg of VGB3 on alternate days and the control group received PBS i.v. into the mice for 19 days from day 13. The tumor volume was measured every 5 days by a digital Vernier caliper (Mitutoyo, Japan), using the following formula: v=a^2^×b×0.52 (where a is the shortest and b is the longest diameter). All studies were performed under the guidelines and with the approval of the Institutional Animal Care and Use Committee (IACUC) of Tehran University of Medical Sciences.

2.14 Immunohistochemistry

Excised tumor tissues for immunohistochemical (IHC) analysis were fixed in formalin (4%), embedded in paraffin, sectioned, and stained with hematoxylin-eosin (H&E). IHC was followed by heat-induced epitope retrieval in a buffer at pH 9.0. IHC staining for CD31 and CD34 (to assign MVD), Ki67 (to determine the percentage of Ki67-positive cells), and P53 and Bcl-2 (to determine the fraction of apoptotic cells relative to total cells) was performed on formalin-fixed paraffin-embedded sections. Staining for cell death with TUNEL (terminal deoxynucleotidyl transferase nick-end labeling)) was performed, followed by enzymatic development in diaminobenzidine (DAB) (Invitrogen, Carlsbad, California, USA) detection and counter-staining in hematoxylin. Images were acquired by microscopy (Olympus BX-51, Japan). Numbers of positive cells were determined by analyzing five random tissue samples under scale bars 100 and 20 μm, with quantification using ImageJ.

2.15 Statistical analysis

The Prism software (version 6.00 for Windows, GraphPad Software, La Jolla, California, USA; www.graphpad.com) was used for data analysis, for the generation of graphs, and for statistical analysis. Data were provided as mean ± SEM. One-way ANOVA followed by Tukey’s post-hoc test was used to statistical significance for multiple comparisons and Two-way repeated measure ANOVA followed by Tukey’s post-hoc test was used for therapeutic efficacy in affecting tumor growth. P<0.05 was considered significant.

References:

1 [Shibuya](https://www.ncbi.nlm.nih.gov/pubmed/?term=Shibuya%20M%5BAuthor%5D&cauthor=true&cauthor_uid=22866201) M. Vascular Endothelial Growth Factor (VEGF) and Its Receptor (VEGFR) Signaling in Angiogenesis. [Genes Cancer](https://www.ncbi.nlm.nih.gov/pmc/articles/PMC3411125/). 12, 1097–1105 (2011).

2 [Iyer](https://www.ncbi.nlm.nih.gov/pubmed/?term=Iyer%20S%5BAuthor%5D&cauthor=true&cauthor_uid=20501651) S, [Darley](https://www.ncbi.nlm.nih.gov/pubmed/?term=Darley%20PI%5BAuthor%5D&cauthor=true&cauthor_uid=20501651) PI, [Acharya](https://www.ncbi.nlm.nih.gov/pubmed/?term=Acharya%20KR%5BAuthor%5D&cauthor=true&cauthor_uid=20501651) KR. Structural insights into the binding of vascular endothelial growth factor-B by VEGFR-1D2 recognition and specificity, J Biol Chem. 285, 23779-23789 (2010).

3 [Wiesmann C](https://www.ncbi.nlm.nih.gov/pubmed/?term=Wiesmann%20C%5BAuthor%5D&cauthor=true&cauthor_uid=9393862). *et al*. Crystal structure at 1.7 A resolution of VEGF in complex with domain 2 of the Flt-1 receptor. [Cell.](https://www.ncbi.nlm.nih.gov/pubmed/9393862) 5, 695-704 (1997).

4 [Kayikci M](https://www.ncbi.nlm.nih.gov/pubmed/?term=Kayikci%20M%5BAuthor%5D&cauthor=true&cauthor_uid=29335563). *et al*. Visualization and analysis of non-covalent contacts using the Protein Contacts Atlas. [Nat Struct Mol Biol.](https://www.ncbi.nlm.nih.gov/pubmed/29335563) 25, 185-194 (2018).

 5 Wang L. *et al*. Identification of Peptidic Antagonists of Vascular Endothelial Growth Factor Receptor 1 by Scanning the Binding Epitopes of Its Ligands. J. Med. Chem. 60, 6598−6606 (2017).

 6 Sadremomtaz A. *et al*. Dual blockade of VEGFR1 and VEGFR2 by a novel peptide abrogates VEGFdriven angiogenesis, tumor growth, and metastasis through PI3K/ AKT and MAPK/ERK1/2 pathway. Biochim Biophys Acta Gen Subj. 1862, 2688–2700 (2018).

7 Sadremomtaz A. *et al*. Suppression of migratory and metastatic pathways *via* blocking VEGFR1 and VEGFR2. [J Recept Signal Transduct Res.](https://www.ncbi.nlm.nih.gov/pubmed/30929546) 38, 432-441 (2018).

8 Farzaneh Behelgardi M. *et al*. A peptide mimicking the binding sites of VEGF-A and VEGF-B inhibits VEGFR-1/-2 deriven angiogenesis, tumor growth and metastasis. Sci Rep. 8, 17924–17937 (2018).

9 [Assareh](https://www.ncbi.nlm.nih.gov/pubmed/?term=Assareh%20E%5BAuthor%5D&cauthor=true&cauthor_uid=30700502) E. *et al*. A cyclic peptide reproducing the α1 helix of VEGF-B binds to VEGFR-1 and VEGFR-2 and inhibits angiogenesis and tumor growth. [Biochem J.](https://www.ncbi.nlm.nih.gov/pubmed/30700502) 476, 645-663 (2019).

10 Baek YY. *et al*. Arg-Leu-Tyr-Glu tetrapeptide inhibits tumor progression by suppressing angiogenesis and vascular permeability via VEGF receptor-2 antagonism. Oncotarget. 8,11763–11777 (2017).

11 Hamdan F, Bigdeli Z, Asghari SM, Sadremomtaz A, Balalaie S. [Synthesis of modified RGD‐Based peptides and their in vitro activity](https://onlinelibrary.wiley.com/doi/abs/10.1002/cmdc.201800704). ChemMedChem. 14, 282-288 (2019).
